# Characterizing the plant functional traits of coffee agroecosystems in Indonesia

**DOI:** 10.3389/fpls.2025.1743035

**Published:** 2026-01-19

**Authors:** Tin W. Satriawan, Xiangzhong Luo, Liyao Yu, Shafira Nur Ramdhania, Luri Nurlaila Syahid, Meine van Noordwijk, Kurniatun Hairiah, Rika Ratna Sari, Endah Sulistyawati, Massimo Lupascu, Noviana Budianti

**Affiliations:** 1 Department of Geography, National University of Singapore, Singapore, Singapore; 2 Independent Researcher, Bandung, Indonesia; 3 Plant Production Systems, Wageningen University and Research, Wageningen, Netherlands; 4 Department of Soil Science, Faculty of Agriculture, Universitas Brawijaya, Malang, Indonesia; 5 School of Life Sciences and Technology (SITH), Institut Teknologi Bandung (ITB), Bandung, Indonesia

**Keywords:** agroforestry, *Coffea* sp., ecophysiology, leaf economic spectrum, photosynthesis

## Abstract

Indonesia, the world’s third-largest coffee producer, is rapidly expanding coffee agroecosystems, often at the expense of deforestation. Understanding the ecophysiology of coffee agroecosystems is thus critical for assessing their impacts on the regional carbon cycle. However, current knowledge of coffee ecophysiology is largely derived from studies in Central and South America and equatorial Africa, with few observations from Indonesia despite its distinct climatic context and large area. In this study, we measured plant functional traits (i.e., leaf structural, physiological, and chemical traits) of coffee plants at four distinct sites in Java, Indonesia, to assess the spatio-temporal variations of coffee leaf traits along with their relationship with shade and reproductive output. We found that physiological traits showed the largest within-site variation, while structural traits varied most strongly among sites. Across seasons, photosynthetic traits (i.e., light-saturated photosynthetic rate *A_max_* and maximum carboxylation rate *V_cmax_*) exhibited pronounced seasonality at a robusta (*C. canephora*) coffee site, whereas arabica coffee (*C. arabica*) and hybrid (*C. arabica* x *C. canephora*) sites showed greater seasonal shifts in structural traits. We also found that denser shade promoted resource-acquisitive strategies (higher photosynthetic capacity, lower leaf mass per area), but this did not translate into greater fruit production. Our study provides one of the first field-based assessments of the ecophysiology of coffee agroecosystems in Indonesia, which will advance our understanding of coffee expansion on the regional carbon cycle.

## 1 Introduction

Coffee is one of the most highly demanded crop commodities in the world, with Indonesia being the third largest producer globally (International Coffee Organization, 2024). Beyond its economic significance, coffee in Indonesia is commonly cultivated within agroforestry systems, where it grows beneath the canopy of shade trees. Intercropping coffee with other species provides ecological and social benefits, such as: (1) enhancing crop resilience by buffering extreme weather and reducing pests and diseases, (2) improving soil fertility and erosion, (3) increasing carbon sequestration and biodiversity, (4) diversifying income for farmers from shade tree products (e.g., fruits, timber, firewood), and (5) providing food security for households (Koutouleas et al., 2022). Thus, these production systems not only sustain livelihoods but also potentially serve as climate mitigation and adaptation strategies (Martin et al., 2020; van Noordwijk et al., 2021; Terasaki Hart et al., 2023). However, when established on previously forested land, coffee agroecosystems may lead to substantial carbon losses (Terasaki Hart et al., 2023). Since 1980, Indonesia’s coffee production area has increased by 80%, from 0.7 million ha to 1.2 million ha in 2021 (Badan Pusat Statistik Indonesia, 2021). Unfortunately, much of this expansion has occurred at the expense of remaining forest (Goldman et al., 2020), resulting in uncertain consequences for regional land-atmosphere CO_2_ exchange. Thus, characterizing the ecophysiology of coffee agroecosystems—including how coffee plants take up, store, and release carbon—is critical for understanding their role in the regional carbon cycle.

Plant functional traits, defined as morpho-physiological or phenological features measurable at the individual level (Violle et al., 2007), are commonly used to examine plant-environment interactions in agricultural contexts (Garnier and Navas, 2012). Leaf traits, including physiological (e.g., maximum photosynthetic carboxylation rate or *V_cmax_*, maximum electron transport rate or *J_max_*), and structural traits (e.g., leaf mass per area or LMA, leaf area index per unit land surface) reflect trade-offs between resource-acquisition and conservation along the leaf economic spectrum (LES). Using a trait-based approach, these individual-level traits can further be upscaled to ecosystem-level processes (Enquist et al., 2017), thus offering important insights into how organisms interact with their environment (Garnier and Navas, 2012). Additionally, field measurement on leaf traits can help inform process-based ecosystem models to simulate crop performance and land surface processes, such as carbon, water, and energy balance (Xu and Trugman, 2021).

In coffee agroecosystems, trait-based approaches have been widely used to investigate the response of coffee plants to climate stresses (DaMatta and Ramalho, 2006; Cavatte et al., 2012), shading (Vaast et al., 2006; Bote and Struik, 2011, 2011; Oliosi et al., 2016), elevation gradients (Getachew et al., 2022), fertilizer treatment (Buchanan et al., 2019), and disease pressures (Gagliardi et al., 2023). For instance, coffee physiological traits were reported to have a high plasticity across light, temperature, and moisture gradients, while chemical traits such as leaf nitrogen (N) concentration are mainly controlled by resource availability (soil biology, soil chemistry) and soil fertility management (Martin et al., 2017; Getachew et al., 2022). In contrast, structural traits such as LMA, leaf dry matter content (LDMC), and leaf thickness showed less variation but more unpredictable responses to environmental fluctuations (Martin et al., 2017; Buchanan et al., 2019). Together, these studies highlight the substantial variation in coffee traits, shaped by both climatic factors and management practices (Gagliardi et al., 2015; Martin et al., 2017; Martin and Isaac, 2021).

Besides varying across growing regions, leaf traits in coffee also vary temporally (Oliosi et al., 2020; Silva et al., 2004). Photosynthetic capacity in arabica coffee, for example, was observed to be higher in the warm and wet seasons in Southeastern Brazil than in the dry seasons (Silva et al., 2004). Meanwhile, leaf chemical traits vary with climatic seasonality (e.g., higher nutrient availability in the wet season) (Santos et al., 2021), and/or phenological phases (e.g., nutrient translocation from the leaves to the fruit during the fruit-filling stage) (Oliosi et al., 2020; Santos et al., 2021; Silva et al., 2022; León-Burgos et al., 2024b).

Although coffee plants have been extensively studied, most of our understanding of their ecophysiology is derived from field studies in Central and South America and equatorial Africa (Melke and Fetene, 2014; Charbonnier et al., 2017; Chinchilla-Soto et al., 2021). Research in Southeast Asia remains limited (Chiarawipa et al., 2025), despite distinct patterns of climate-vegetation seasonal response across the tropics (Uribe et al., 2021). Given the dependency of plant traits on the local environment, it is therefore important to conduct leaf trait measurements for coffee agroecosystems in Indonesia.

In this study, we address this gap by characterizing leaf trait variation in Indonesian coffee agroecosystems, involving *C. arabica, C canephora*, and their hybrids. Specifically, we (1) measured key leaf structural, physiological, and chemical traits across multiple sites representing typical coffee-growing environments in Indonesia, (2) assessed the seasonal pattern of these traits, and (3) explored how leaf traits relate to canopy cover and fruit production. We aim to provide essential baseline data on the spatial and temporal variability of coffee leaf traits in this understudied region.

## 2 Materials and methods

### 2.1 Study sites

**Figure 1:**
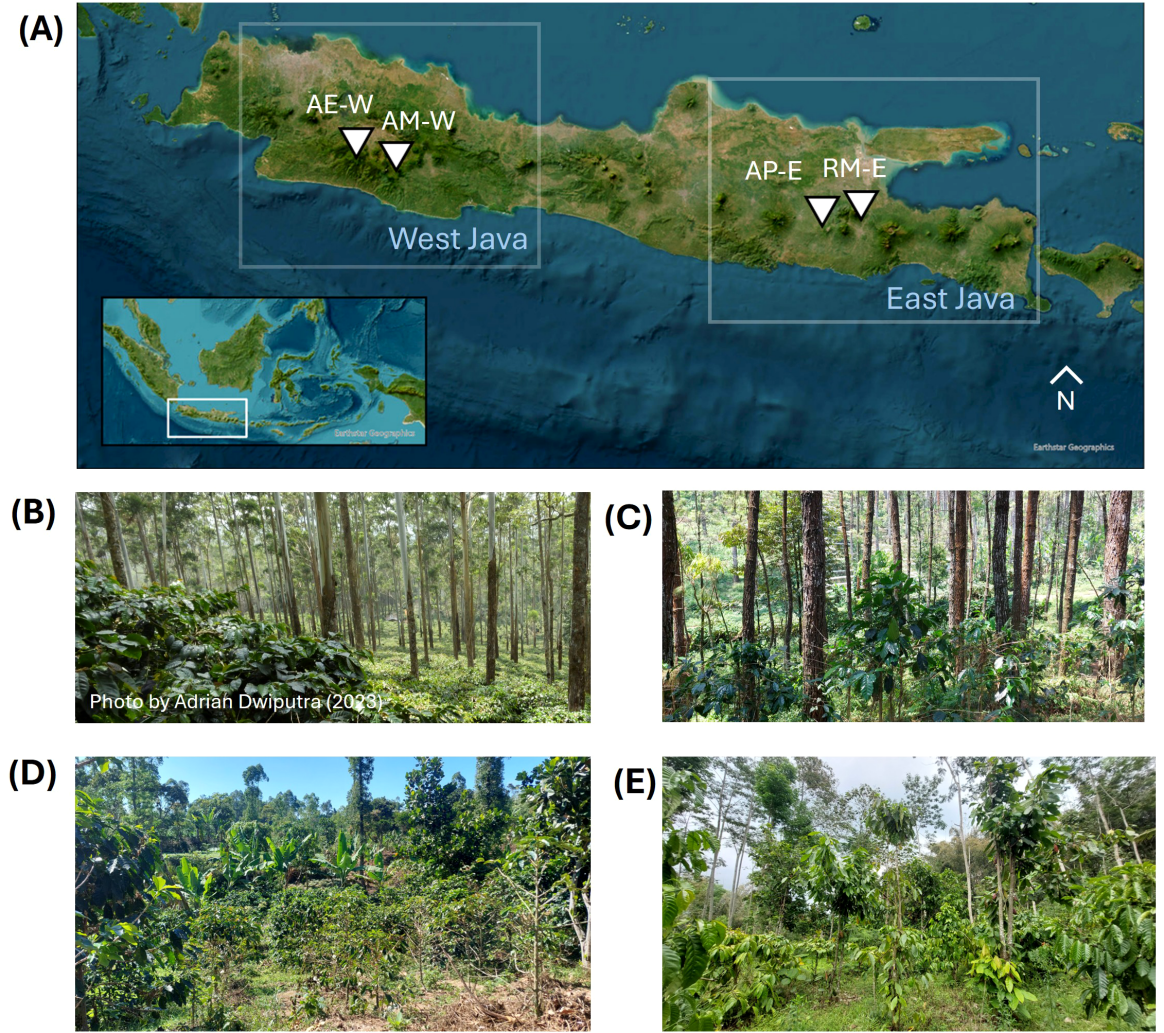
Location of study sites in Java Island **(A)** and the photo of each site taken between November 2023–2024, Indonesia. **(B)** Arabica hybrid–Eucalyptus–West Java or ‘AE-W’ site, **(C)** Arabica–Mixed trees–West Java or ‘AM-W’ site, **(D)** Arabica hybrid–Pine–East Java or ‘AP-E’ site, and **(E)** Robusta–Mixed trees–East Java or ‘RM-E’ site.

Trait data collection was conducted at four coffee plantations in Java Island, Indonesia (**Figure 1**; **Table 1**). The first site (‘AE-W’, for *Arabica hybrid–Eucalyptus–West Java*) is located at Riung Gunung, Pangalengan, Bandung Regency, West Java (7° 9’58.25”S, 107°31’8.47”E). The coffee farm belongs to Java Frinsa Estate and is situated at the foothill of Mount Tilu. The coffee variety grown at this site is Timor hybrid (‘hibrido de timor’), which is a natural cross of arabica and robusta originating from East Timor in the 1920s, known to be resistant to leaf rust. Timor hybrid’s leaf length is typically around 21 cm, with greenish-brown young leaves and dark green mature leaves (Budiasih et al., 2025). The coffee plants are grown at 1 × 3 m spacing together with timber species shade trees with dense canopy (83.4% cover), such as *Eucalyptus* sp. (78 trees/ha; 33 m height) and *Agathis dammara* saplings (78 trees/ha; 4 m height). This site has the highest elevation (1621 m) among all sites, making it the coolest and wettest site. This site is also the most intensely managed, where it receives around 750 kg/ha of nitrogen-phosphorus-potassium (N-P-K) fertilization and 500 kg/ha of dolomite per year, and is regularly pruned to *ca.* 1.75* m* height and weeded after the harvesting season.

**Table 1:** The characteristics of four sites in Java, including coffee planted, shade trees, and physical environment.

Site variable	AE-W	AM-W	AP-E	RM-E
Coffee type	‘Timor’ (*C. arabica* L. *× C. canephora*)	‘Typica’ Garut (*C. arabica* L.)	‘Catimor’, a cross between Caturra (*C. arabica* L.) and Timor (*C. arabica × C. canephora*)	*C. canephora* Clone BP
Coffee plant height (mean ± SE)	176.03 ± 2.99 cm	179.14 ± 8.26 cm	152.33 ± 3.6 cm	144.18 ± 4.72 cm
Coffee stem diameter (mean ± SE)	8.38 ± 0.26 cm	5.7 ± 0.54 cm	5.95 ± 0.36 cm	18.78 ± 1.13 cm
Coffee plant age	9 years old	6 years old	8 years old	10 years old
Shade trees	*Eucalyptus* sp.*, Agathis dammara*	*Citrus* sp., *Artocarpus heterophyllus*	*Pinus radiata*, *Pinus merkusii*	*Durio zibethinus*, *Theobroma cacao*, *Syzygium aromaticum*, *Persea americana*
Canopy cover (mean ± SE)	83.39 ± 1.37 %	31.18 ± 6.13 %	94.03 ± 0.52 %	71.34 ± 4.29 %
Elevation	1621 m a.s.l.	1440 m a.s.l.	1251 m a.s.l.	725 m a.s.l.
Management	Fertilized multiple times (post-harvesting and fruit-filling phase), pruned at 1.5 m	Fertilized once (post-harvesting), not pruned	Not fertilized, rejuvenized	Fertilized once (post-harvesting), pruned at 1.5 m
Temperature at 15 cm	17.88 °C Range: 13.77 – 25.64 °C	20.52 °C Range: 13.56 – 27.44 °C	20.36 °C Range: 15.18 – 24.76 °C	23.18 °C Range: 18.18 – 29.18 °C
Soil moisture	41.79% Range: 23.67 – 50.1%	39.96 % Range: 23.30 – 52.0 %	39.85% Range: 23.4 – 48.7%	39.05% Range: 23.7 –50.6%
Soil type	Ochric andosols	Humic andosols	Mollic andosols	Ochric andosols
Precipitation	4052 mm/year Range: 44.74 – 744.50 mm/month	3673 mm/year Range: 21.72 – 682.32 mm/month	3174 mm/year Range: 12.92 – 755.76 mm/month	3382 mm/year Range: 14.40 – 693.84 mm/month

For the basis of range in temperature, soil moisture, and precipitation during the period of measurement, see Section 2.2. Soil types are based on FAO-Unesco (1979).

The second site (‘AM-W’, for *Arabica–Mixed trees–West Java*) is located at Pasir Sereh, Cisurupan District, Garut Regency, West Java (7°17’28.44”S, 107°45’56.25”E), which is situated on the foothill of Mount Papandayan. This site grows arabica var. Typica, which was historically originated from in Malabar, Java in the 18^th^ century. This variety has low yield and is less resistant to leaf rust, but is adapted to high elevation (World Coffee Research, 2025). Arabica var. Typica generally has a leaf length of around 16* cm* and dark green-colored mature leaves, and could naturally grow up to 3–5 m (Budiasih et al., 2025). Coffee plants at this site are grown together with mixed fruit tree species as shade trees, such as citrus (133 trees/ha; 3.44 m height), jackfruit (48 trees/ha; 5.22 m height), and mango (26 trees/ha; 16.55 m height). It is characterised by a reduced shade (31% canopy cover) and minimum management measures, where coffee plants are not pruned but given organic fertilizer once or twice a year during the flowering period. Further details on shade tree characteristics are provided in **Supplementary Materials**, **Supplementary Figure 3**.

The third site (‘AP-E’, for *Arabica hybrid–Pine–East Java*) is located at Brawijaya University education forest (UB Forest), Sumbersari, Karangploso, Malang Regency, East Java (7°49’28.10”S, 112°34’29.06”E) at the foothill of Mount Arjuna. This site is characterized by the dense canopy (94% cover) of pine trees (*ca.* 800 trees/ha) with ‘Catimor’ coffee growing underneath. ‘Catimor’ variety is a cross between Caturra (*C. arabica*) and Timor (*C. arabica* x *C. canephora*), and is resistant to coffee leaf rust and berry diseases (World Coffee Research, 2025). This variety generally has a leaf dimension of 18.6 × 7.1 cm, with plant height around 140 cm (Budiasih et al., 2025). Coffee plants at this site were recently rejuvenated, but with low management intensity.

The fourth site (RM-E, for *Robusta–Mixed trees–East Java*) is located in Jabon village, Ngantang District, Malang Regency, East Java (7°50’39.99”S, 112°22’48.06”E). Coffee plants grown here are robusta clone of BP (Besoekisch Proefstation), which is one of the national superior clones suitable for areas with elevation along 400–900 m. BP clones have green-colored elliptical leaves with wavy surface, with a length distinctively larger than arabica leaves of *ca.* 24 cm (Ramadiana et al., 2018). This site is characterized by robusta coffee grown under moderate canopy (71% cover) of mixed fruit and mixed timber shade trees, such as durian (81 trees/ha, tree height 5.81 m), avocado (74 trees/ha, tree height 6.57 m), cacao (85 trees/ha, 4.12 m). This site is situated at a lower elevation (725 m) and has a higher mean temperature compared to other sites. The management intensity is medium, where coffee plants are pruned and given organic fertilizer once or twice a year. At each site, we set up three plots with a size of approximately 30 × 30 m^2^. Within each plot, we randomly chose five healthy coffee plants (i.e., no visible damage and minimum discoloration of leaves) to measure their functional traits. To capture the seasonality, we conducted a round of measurement in the dry season (September – October 2024) and another round in the wet season (February – March 2025), which coincides with the flowering and the fruit-filling period, respectively. The measurement was conducted over a period of one week per site and season. For the AE-W site, four individuals were cut for thinning in the middle of the study period, so we replaced them with the neighbouring individual for the wet season measurement.

### 2.2 Environmental and ancillary data

At each plot, we installed a Temperature-Moisture-Sensor (TMS) datalogger (TOMST, Prague), which measures air temperature at 15 cm above the soil surface (*T_a,15cm_*), surface temperature, and soil temperature and moisture at 8 cm depth from September to March 2025. Unfortunately, all three TMS dataloggers at the AE-W site and one at AM-W went missing, and their data could not be retrieved. To address these gaps, we estimated missing environmental variables using ERA5-Land hourly data (Muñoz-Sabater, 2019) for the period 1 April 2024 to 31 March 2025. ERA5-Land is a global gridded land-surface dataset produced by the European Centre for Medium-Range Weather Forecasts (ECMWF) at 0.1° spatial resolution. We specifically estimated:

- Air temperature at 15 cm (Ta, 15cm) using a linear regression model between daily observed Ta,15cm and ERA5-Land 2 m air temperature at each site. Site-specific models were trained using days with complete data and applied to predict missing values.
- Soil moisture at 8 cm using a Random Forest model trained on days with observed TMS data. Predictor variables included ERA5-Land-derived soil volumetric water content (0–7 cm layer), precipitation, and surface temperature.

### 2.3 Functional traits data collection

To measure leaf physiological traits, we selected one healthy, sunlit, fully expanded leaf from the upper 30% of the plant canopy to minimize variation associated with leaf position and uneven sun exposure throughout the day. Physiological trait measurements were conducted on site on one attached leaf from five coffee plants per plot. We measured light response curves (LRCs) and CO_2_ response curves (*A/C_i_*) using a portable gas exchange system Li-6800 (LiCor, Lincoln, NE, USA). LRC was recorded under a reference CO_2_ concentration of 400 μmol mol^-1^, at a leaf temperature of 25 °C, and relative humidity (RH) between 50–60%. To build LRCs, we measured CO_2_ assimilation rate (*A*) under 14 decreasing steps of photosynthetic photon flux density (PPFD), at 1200, 1000, 800, 700, 600, 500, 400, 350, 300, 250, 200, 150, 100, 50, 0 µmol m^−2^ s^−1^ following (Xiao et al., 2024). From the LRCs, we inferred dark respiration (*R_d_*), light-saturated photosynthetic rate per unit area (*A_max_*), light-saturated photosynthetic rate per unit dry mass (*A_mass_*), transpiration (*E*), and stomatal conductance (*g_sw_*) at saturating PPFD and calculated intrinsic water use efficiency (iWUE) as *A_max_*/*g_sw_*, and instantaneous water use efficiency (WUE) as *A_max_*/*E*. We also derived photosynthetic quantum yield (α) by fitting an exponential model to the LRCs (Bassman and Zwier, 1991; He et al., 2024).

When measuring *A*/*C_i_* curves, we set the PPFD to 800 µmol m^-2^ s^-1^, where photosynthesis of most coffee plants appears to saturate without signs of photoinhibition. We then measured *A* at 15 steps of reference CO_2_ of 400, 300, 200, 100, 150, 250, 350, 400, 450, 550, 650, 750, 850, 950, 1100 μmol mol^-1^. In total, we obtained 60 coffee leaf LRCs and 60 *A/C_i_* curves for each season (note: 55 *A/C_i_* curves for the wet season due to equipment malfunction). Using the ‘Plantecophys*’* package in R (Duursma, 2015), we estimated photosynthetic parameters, maximum carboxylation rate (*V_cmax_*)and maximum electron transport rate (*J_max_*), by fitting the Farquhar-von Caemmerer-Berry (FvCB) model of leaf photosynthesis (Farquhar et al., 1980) to the *A/C_i_*curves.

After collecting the leaf physiological traits data, we sampled the measured leaf along with four additional leaves from the same plant to measure their structural traits. We measured the leaf thickness, leaf wet mass without the petiole, and leaf area. Leaf thickness was measured using a 0–25 mm digital micrometer (Mitutoyo, IL, USA) averaged across three locations of the leaf. To obtain leaf area, we scanned the leaf using a digital scanner and estimated the area using ImageJ software (Schneider et al., 2012). We then dried the leaves in the oven at 70–80 °C for 84 hours, recorded their dry mass, and calculated their LMA as leaf dry mass divided by leaf area, and leaf dry matter content (LDMC) as dry mass divided by wet mass.

The dry leaves were then ground for further chemical analysis. Leaf carbon (C) content, leaf nitrogen (N) content, C isotope (δ^13^C), and N isotope (δ^15^N) were analysed using the Elemental Analyzer-Isotope Ratio Mass Spectrometry (EA-IRMS) technique (EA: EuroVector, model EA3000; IRMS: Nu Instruments, model Perspective). In total, we measured the total C and N content of 300 coffee leaves for each season. We also analysed leaf phosphorus (P) content, but only for the 60 leaves that were measured for their physiological traits. For P analysis, we conducted acid digestion using HNO_3_ on the dry ground leaves. P contents were then analysed using Inductively Coupled Plasma Optical Emission Spectroscopy (ICP-OES) (Agilent).

In addition to leaf traits, we measured plant height, stem diameter at 15 cm above ground, canopy cover above the plant, and the number of fruits per plant. The canopy cover above the coffee plant was measured using a densiometer (0 – 100%, with higher percentages having more shade). Fruit counts were estimated following the protocol described by Cilas and Descroix (2004) using a multiplicative method based on plant architecture:

Fruit count per plant=(number of stems per plant)×(fruit bearing branches per stem)×(glomerules per branch)×(fruits per glomerule)

For each plant, the number of stems was counted directly. One representative stem was selected per plant to assess reproductive architecture. On this stem, we counted the number of fruit-bearing branches, then randomly selected four of these branches to estimate the number of glomerules per branch. On each of the four branches, two glomerules were randomly chosen (eight glomerules per plant in total) to count the number of fruits per glomerule.

### 2.4 Statistical analysis

For each plant, leaf traits were averaged across sampled leaves to obtain individual-level trait estimates. For site-level comparisons, we further averaged these individual values across the two sampling seasons (wet and dry) to derive a single site-level trait mean. Descriptive statistics (mean and (plant-level) standard error) were then calculated for each trait at each site. Trait variability was quantified using the coefficient of variation (CV) calculated at two scales: (1) the overall CV across all plants in the dataset, and (2) the site-level CV based on plants within each site. To test whether site-level trait variation differed significantly from overall trait variation, we used a null model approach based on a bootstrapping procedure. Specifically, we generated 500 randomized datasets following the method described in Buchanan et al. (2019) and compared observed site-level CVs against the distribution of bootstrapped values. To assess seasonal effects on traits, we compared within-site trait means between wet and dry seasons using a one-way analysis of variance (ANOVA), followed by a *post-hoc* pairwise T-test. All statistical analysis was carried out using R 4.3.2 (R Core Team, 2023).

To examine the coordination of leaf traits, we conducted a principal component analysis (PCA) using ‘prcomp’ function within the ‘stats’ package using the plant-level data aggregated across sites and seasons. We excluded traits that are highly correlated with each other (**Supplementary Material**, **Supplementary Figure 2**). Thus, the traits used in the PCA were LMA, LDMC, *V_cmax_*, *A_mass_*, *g_sw_*, leaf N, leaf C, leaf K, and leaf P. We then evaluated the significance of the PCA by comparing the observed correlation structure to that expected by chance (Björklund, 2019) using the ‘PCAtest’ package (Camargo, 2025). Using the resulting two principal components (PCs), we analysed the relationship between trait strategies, canopy cover, and reproductive output. We fitted the scores of the two PCs to canopy cover and fruit count using a linear model. We tested the linear model with and without season as an interaction term using analysis of variance (ANOVA).

We also assessed pairwise correlations among leaf traits and compared these relationships with those reported in other published datasets. Specifically, we queried the TRY plant trait database (Kattge et al., 2019) for records of *Coffea arabica* and *Coffea canephora* that included at least one combination of LMA, leaf N, and *A_mass_*. The search resulted in three datasets (Martin et al., 2017; Buchanan et al., 2019; Gagliardi et al., 2023). For each trait pair, we then fitted a linear model across all datasets, including ours, and included dataset identity as an interaction term to test whether trait relationships differed among studies.

## 3 Results

### 3.1 Environmental variables across coffee agroecosystems

**Figure 2:**
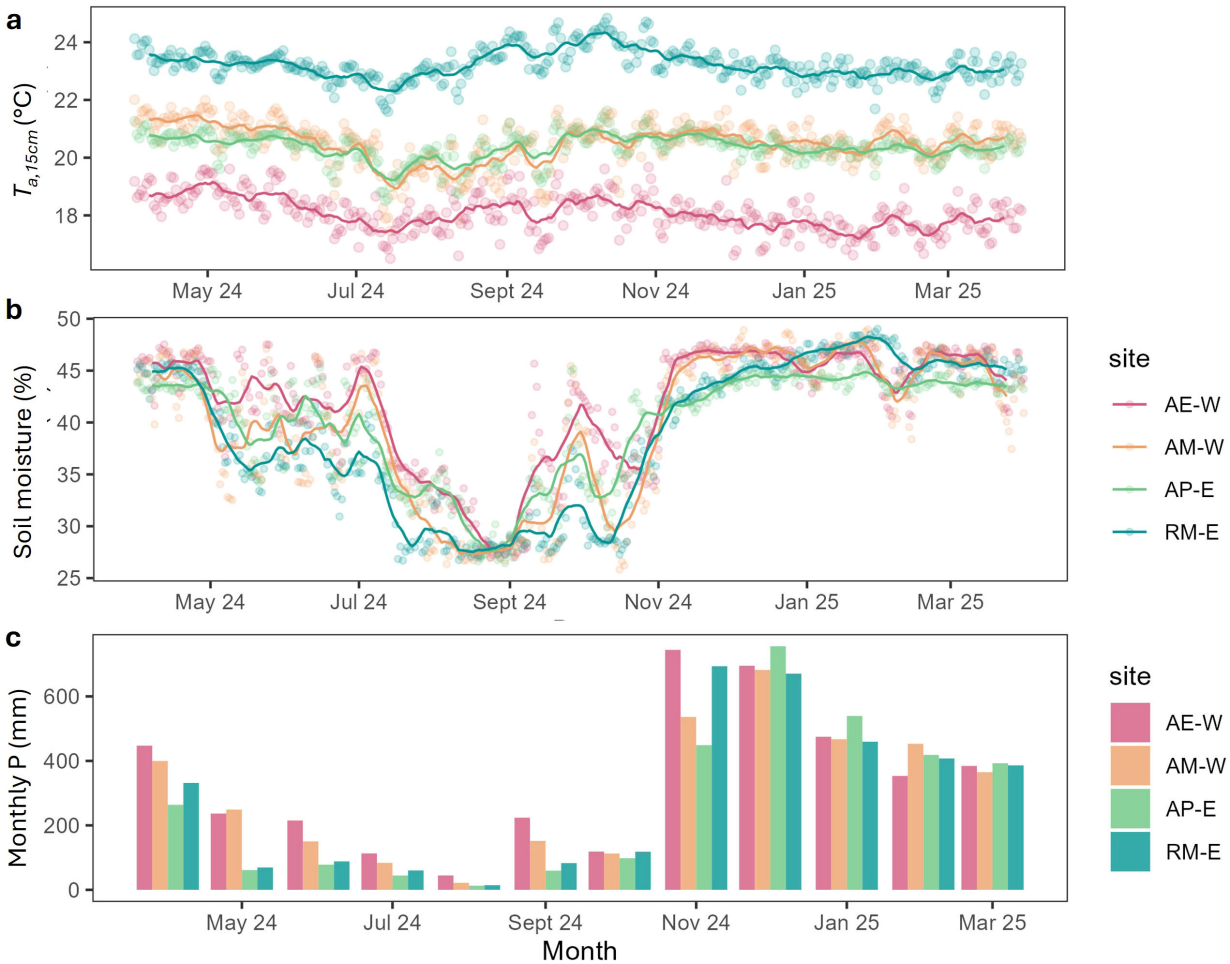
Seasonal pattern of climatic variables at different sites from April 2024 to March 2025 **(a)** understory air temperature at 15 cm above the surface (T_a,15cm_), modelled from ERA-5 air temperature and measurements from dataloggers, **(b)** soil moisture modelled from ERA-5 and measurements from dataloggers, **(c)** monthly precipitation (P) from ERA-5. Data points indicate daily measurements, and lines indicate 16-day rolling mean.

The four sites showed clear variation in environmental conditions (**Figure 2**). AE-W was the coolest and wettest among all sites, with a mean T_a,15cm_ of 17.88 °C on average (range: 13.77 – 25.64 °C), and total precipitation of 4052 mm/yr during the study period. AM-W experienced similar annual precipitation (3673.7 mm/yr), but a higher average T_a,15cm_ of 20.52 °C (range: 13.56 – 27.44 °C) than AE-W. In contrast, the two East Java sites (AP-E and RM-E) were at lower elevations and received less annual precipitation—3174mm/yr and 3382 mm/yr, respectively. Among them, AP-E had a cooler T_a,15cm_ (20.36 °C, range: 15.18 – 24.76 °C). RM-E was the warmest site overall, with a mean T_a,15cm_ of 23.18 °C (range: 18.18 – 29.18 °C).

Seasonal variation in temperature was relatively weak at all sites, while precipitation and soil moisture showed stronger seasonality. AP-E and RM-E experienced a pronounced dry period from May to October, in which the minimum monthly precipitation reached 13 mm/month for AP-E and 14 mm/month for RM-E, compared to the wetter minimum monthly precipitation in West Java (AE-W: 44.74 mm, AP-W: 21.72 mm). However, despite the lower rainfall, AP-E retained soil moisture more effectively during the dry season. Overall, the two less-shaded sites (AM-W and RM-E) exhibited larger seasonal variations in soil moisture, while the two shaded sites had weaker seasonality, reflecting the buffering effect of shade trees, regardless of the amount of precipitation they receive.

### 3.2 Coffee leaf trait variation within and across sites

**Figure 3:**
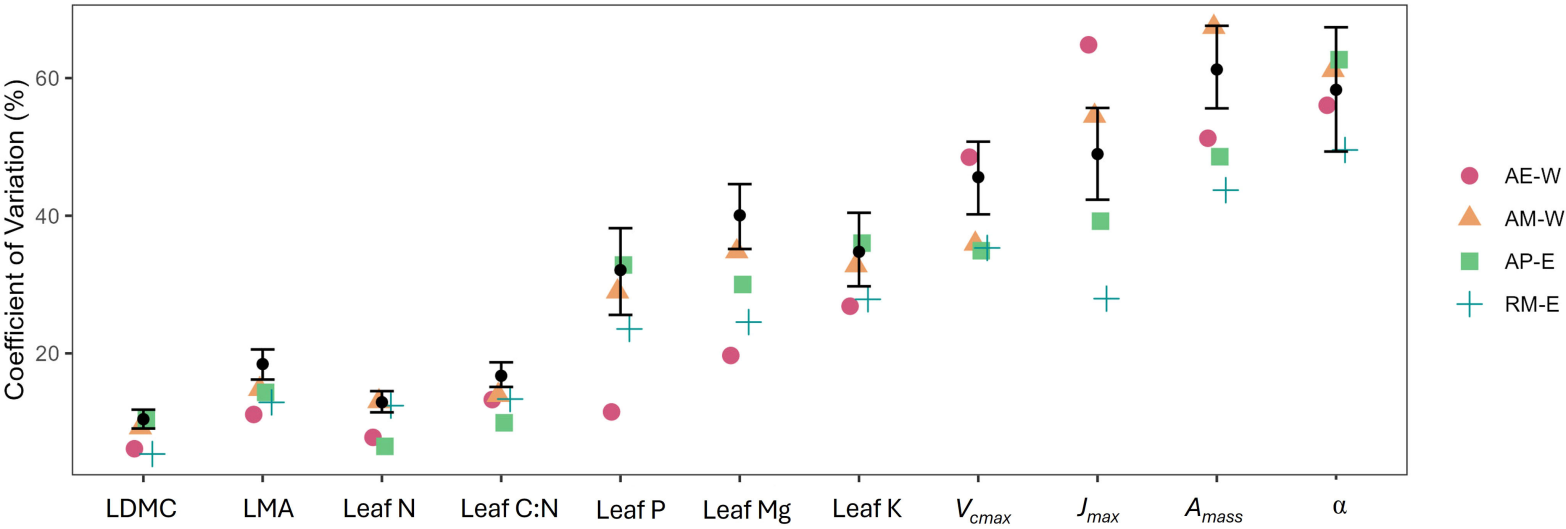
Leaf traits variation across different sites calculated as the coefficient of variation (CV) in %. The black dot represents the overall CV, and the bars indicate the 95% CI estimated from bootstrapping. When the CV values fall outside the 95% CI, the null hypothesis of pure random trait variation is rejected.

We examined site-level means (**Table 2**; **Supplementary Material**, **Supplementary Figure 3**), as well as the extent of variation in coffee leaf traits among coffee plants of all sites (overall CV) and within each site (site-level CV) (**Figure 3**). We found that structural traits showed low within-site variation but clear site differences in trait means, while chemical and physiological traits showed larger within-site variation with limited between-site differences in trait means.

**Table 2:** Descriptive statistics (mean ± standard error) of leaf structural, chemical, and physiological traits averaged across seasons (n = 60 individuals) for each site.

Traits	AE-W	AM-W	AP-E	RM-E
Leaf structural traits				
Leaf area (cm^2^)	64.14 ± 1.19 ^b^	47.19 ± 1.72 ^c^	65.69 ± 3.18 ^b^	194.55 ± 4.63 ^a^
Leaf thickness (mm)	0.296 ± 0.003 ^b^	0.322 ± 0.005 ^a^	0.284 ± 0.004 ^b^	0.229 ± 0.005 ^c^
LDMC (g g^−1^)	0.357 ± 0.003 ^a^	0.368 ± 0.007 ^a^	0.31 ± 0.007 ^b^	0.369 ± 0.005 ^a^
LMA (g m^−2^)	94.7 ± 1.49 ^b^	113.27 ± 3.05 ^a^	81.11 ± 2.64 ^c^	87.37 ± 2.79 ^bc^
Leaf chemical traits				
d15N	2.65 ± 0.17 ^a^	5.28 ± 0.28 ^b^	0.6 ± 0.26 ^c^	-0.81 ± 0.27 ^d^
Δ	21.43 ± 0.11 ^a^	18.85 ± 0.17 ^b^	22.94 ± 0.15 ^c^	22.4 ± 0.23 ^c^
δ^13^C	-28.81 ± 0.1 ^a^	-26.36 ± 0.16 ^b^	-30.24 ± 0.15 ^c^	-29.73 ± 0.22 ^c^
Leaf N (% dry mass)	2.58 ± 0.04 ^a^	2.62 ± 0.06 ^a^	3.07 ± 0.03 ^b^	2.61 ± 0.08 ^a^
Leaf C (% dry mass)	55.43 ± 0.28 ^b^	57.78 ± 0.17 ^a^	52.46 ± 0.27 ^c^	56.19 ± 0.33 ^b^
C:N	21.84 ± 0.37 ^a^	22.5 ± 0.59 ^a^	17.26 ± 0.22 ^b^	21.88 ± 0.6 ^a^
Leaf P (% dry mass)	0.123 ± 0.003 ^a^	0.143 ± 0.009 a	0.177 ± 0.012 ^b^	0.119 ± 0.004 a
Leaf Mg (% dry mass)	0.29 ± 0.01 ^a^	0.49 ± 0.04 ^b^	0.27 ± 0.02 ^a^	0.44 ± 0.02 ^b^
Leaf K (% dry mass)	1.59 ± 0.09 ^ab^	1.32 ± 0.1 ^b^	1.78 ± 0.12 ^a^	1.36 ± 0.09 ^b^
Leaf physiological traits				
*V_cmax_*(µmol CO_2_ m^2^ s^−1^)	22.1 ± 2.17^b^	21.14 ± 1.76^b^	39.26 ± 2.63^a^	35.91 ± 2.17^a^
*J_max_*(µmol m^2^ s^−1^)	39.16 ± 6.68^b^	39.63 ± 5.33^b^	79.43 ± 7.11^a^	74.25 ± 4.07^a^
*A_max_* (µmol CO_2_ m^2^ s^−1^)	4.26 ± 0.37^b^	4.95 ± 0.43^b^	7.25 ± 0.7^a^	7.75 ± 0.42^a^
*A_mass_* (µmol CO_2_ g s^−1^)	0.046 ± 0.004 ^b^	0.047 ± 0.005 ^b^	0.092 ± 0.01 ^a^	0.087 ± 0.006 a
α (mol CO_2_ mol photons^−1^)	0.013 ± 0.001	0.012 ± 0.001	0.012 ± 0.002	0.011 ± 0.001
E (mmol H_2_O m^2^ s^−1^)	0.46 ± 0.05 ^b^	0.5 ± 0.04 ^b^	1.13 ± 0.14 ^a^	1.13 ± 0.09 ^a^
*R_d_* (µmol CO_2_ m^2^ s^−1^)	-0.995 ± 0.101 ^b^	-0.939 ± 0.077 ^b^	-0.638 ± 0.075 ^a^	-0.597 ± 0.068 a
*g_sw_* (mol H_2_O m^2^ s^−1^)	0.025 ± 0.003 ^b^	0.027 ± 0.003 ^b^	0.066 ± 0.009 ^a^	0.07 ± 0.006 a
iWUE (mmol CO_2_ mol H_2_O^−1^)	0.153 ± 0.018	0.136 ± 0.009	0.123 ± 0.006	0.114 ± 0.004
WUE (mmol CO_2_ mol H_2_O^−1^)	8.15 ± 0.89	7.62 ± 0.49	7.15 ± 0.35	6.93 ± 0.26

AE-W, arabica hybrid coffee shaded by eucalyptus (West Java); AM-W, arabica coffee shaded by mixed trees (West Java); AP-E, arabica hybrid coffee shaded by pine (East Java); RM-E, robusta coffee shaded by mixed trees (East Java). Small superscript letters indicate significantly different means (p<0.05) following the Tukey HSD test, where groups that differ significantly have different letters and groups that do not differ from each other share the same letter.

Structural traits (e.g., LDMC and LMA) exhibited small within-site variation (site CV<20%), but showed clear differences between sites. In particular, leaf area and dry mass are the largest at the robusta site (RM-E), followed by the hybrids and arabica sites. In contrast, leaf thickness and LMA are the highest at AM-W. Within-site variations in structural traits for all sites are significantly smaller than expected by chance.

In contrast, leaf chemical traits have a large range of within-site variation, in which leaf N and C:N is less varied (site CV< 20%), while leaf P, Mg, and K content are more varied (site CV ~30%). Leaf chemical (N, C:N, and P) shows similarity between three sites (AE-W, AM-W, and RM-E), while AP-E shows greater N and P and lower C:N. Within-site variations in leaf chemical traits are significantly smaller than expected by chance for AE-W.

Leaf physiological traits (*V_cmax_*, *J_max_*, and *A_mass_*) have the largest variation (CV>30%) among all traits. The East Java sites (AP-E and RM-E) have significantly higher *A_mass_*, *A_max_*, *J_max_*, *V_cmax_*, *E*, and *g_sw_*, and lower *R_d_* than the West Java sites (AE-W and AM-W). At the same time, the East Java sites also exhibited significantly smaller within-site variation than expected in most physiological traits, while α and WUE did not differ across sites.

### 3.3 Seasonality of coffee leaf traits

**Figure 4:**
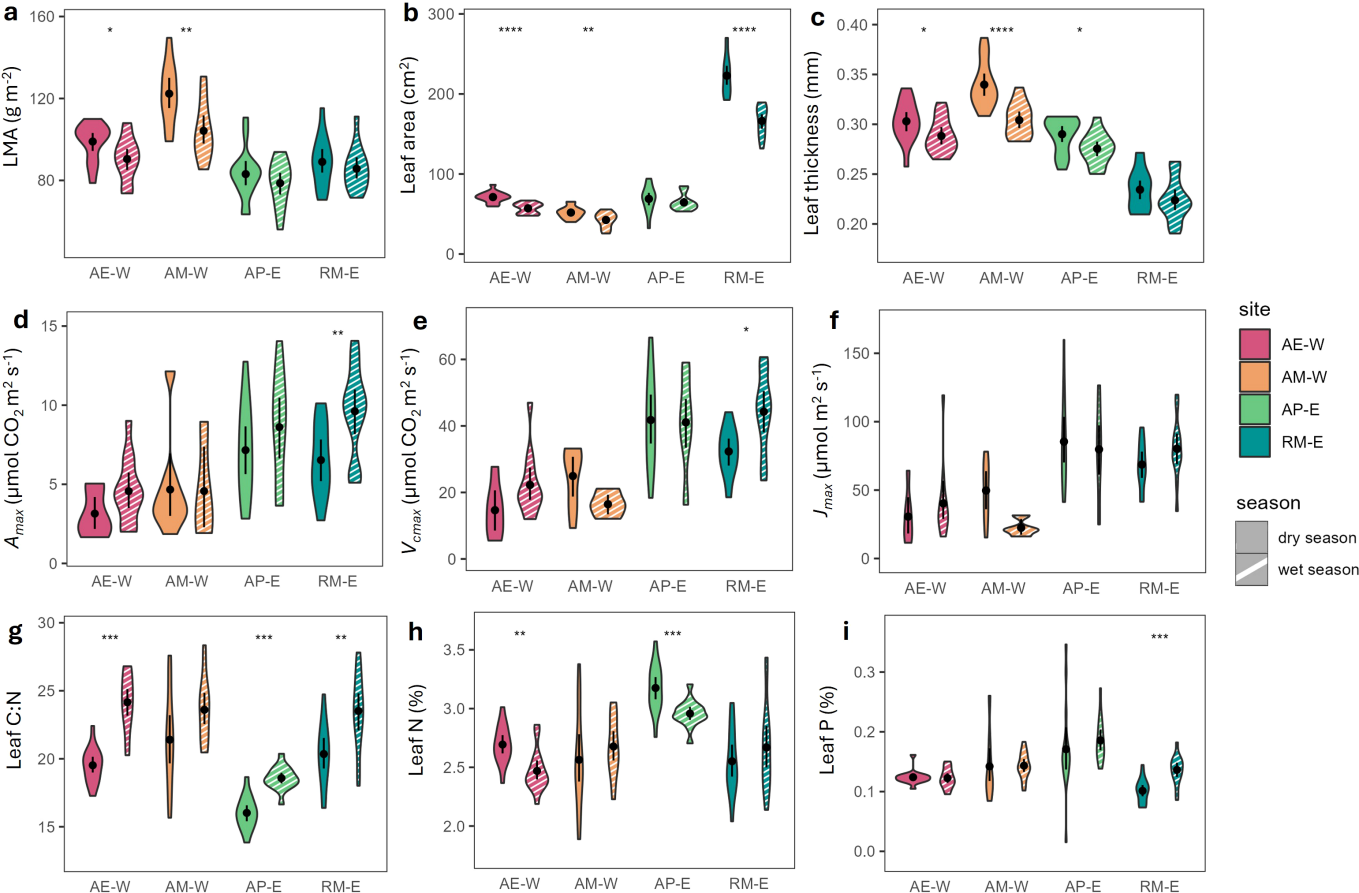
Leaf structural, physiological, and chemical traits across different sites and seasons. **(a)** leaf mass per area (LMA), **(b)** leaf area, **(c)** leaf thickness, **(d)** light-saturated photosynthetic rate per unit leaf dry mass (A_mass_), **(e)** maximum carboxylation rate (V_cmax_), **(f)** maximum electron transport rate (J_max_), **(g)** leaf C:N, **(h)** leaf nitrogen content, and **(i)** leaf phosphorus content. Different colors of the violin plot refer to different sites, and the different patterns refer to different seasons (solid = dry season, white stripe = wet season). The black dot inside the violin plot refers to the mean value, and the error bar represents the confidence interval. T‐test shows the significant difference between the wet and dry seasons within each site, with p‐value< 0.001(****), <0.01(***), <0.05(**), <0.1(*).

We also found that traits vary between dry and wet seasons, although the patterns are not consistent across sites (**Figure 4**). The arabica (AM-W) and hybrid (AE-W and AP-E) sites had similar seasonality in leaf structural and chemical traits, while the robusta coffee site (RM-E) experienced a more pronounced seasonality in physiological traits. In the wet season, AE-W experienced a significant decline in leaf area, leaf thickness, and leaf dry weight (p<0.05). Since the decline in leaf dry mass is larger than that of leaf area at AE-W, it resulted in a significant decrease in LMA. This is accompanied by a lower leaf N and a higher leaf C:N during the wet season. Similarly, AM-W and AP-E also followed this pattern, although AM-W had a significant decline in LMA (p<0.01) with no significant increase in C:N, while AP-E had the opposite pattern. For RM-E, both leaf area and leaf dry mass declined in the wet season, resulting in no significant change in LMA. Instead, RM-E experienced around a 40–50% increase in *A_max_* and *V_cmax_*(p<0.05). While this increase in photosynthetic capacity is not accompanied by an increase in leaf N, we observed an increase in leaf P and leaf C:N at RM-E in the wet season.

### 3.4 Leaf traits coordination

**Figure 5:**
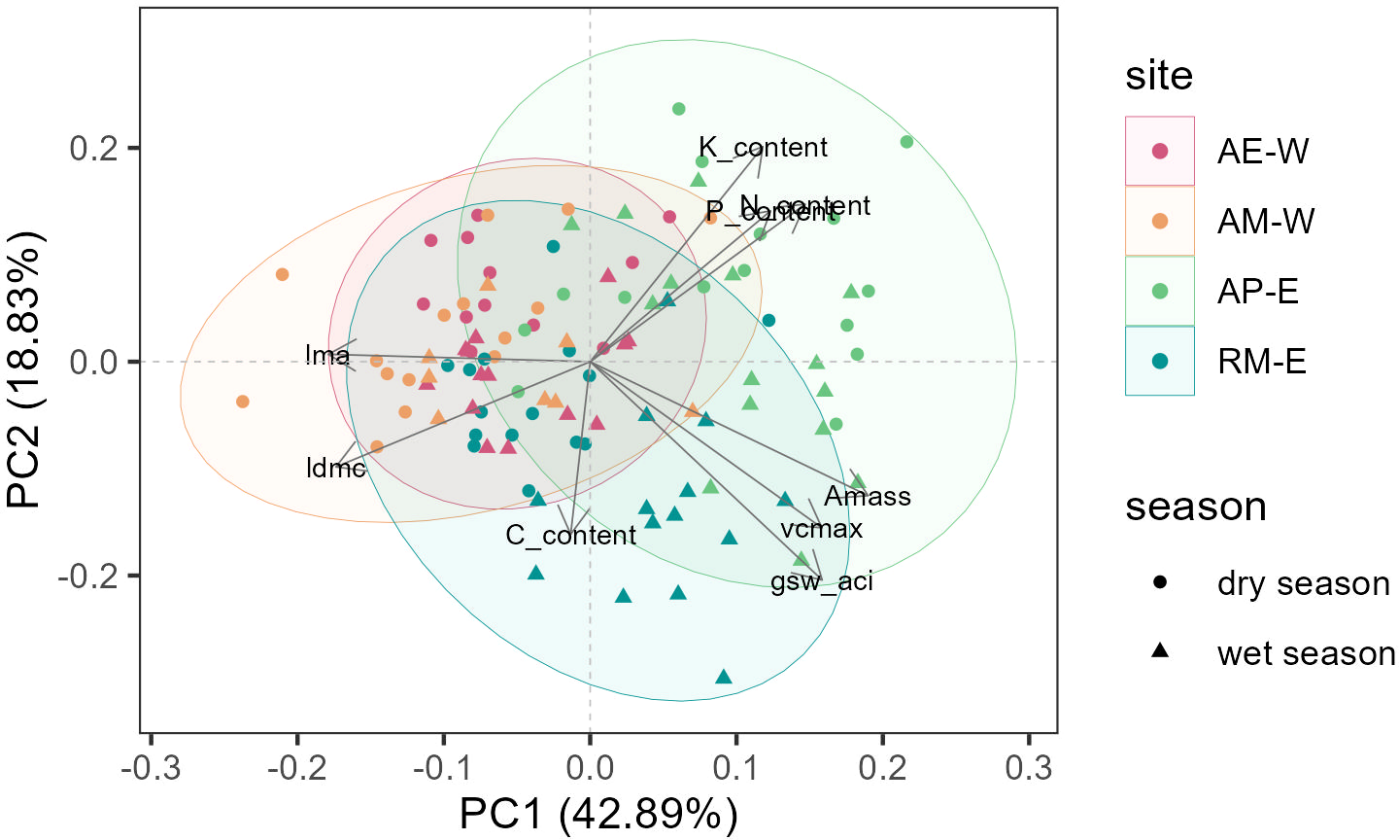
Principal component analysis. The data points refer to leaf trait measurement, where different colors represent different sites, and different shapes represent different seasons (circle for the dry season and triangle for the wet season).

We examined the trait coordination at our sites using PCA of all traits (**Figure 5**), in which the first two principal components (PC) together accounted for 61.72% of the trait variation. PC1 explained 42.89% variation in leaf traits and reflects traits associated with leaf-level carbon gain, where it is positively correlated with *A_mass_*, *V_cmax_*, and *g_sw_*; and is negatively correlated with LMA and LDMC. PC2 explained 18.83% of the variation in leaf traits, and is positively correlated with leaf chemical traits (leaf K, N, P); and is negatively correlated with *g_sw_*, *V_cmax_*, and leaf C.

The two sites in West Java (AE-W and AM-W) overlapped with each other in the two-dimensional trait space, and are characterized by a negative PC1 (high LMA and LDMC; low photosynthetic capacity), although the variation along the PC1 axis is larger for AE-W. In contrast, the arabica site in East Java (AP-E) is characterized by a positive PC1 (high photosynthetic capacity and low LMA). Meanwhile, the robusta site (RM-E) is characterized by a negative PC2 (high *g_sw_* and leaf C), although the variation along this axis is high.

### 3.5 Linking trait strategies to canopy and reproductive output

**Figure 6:**
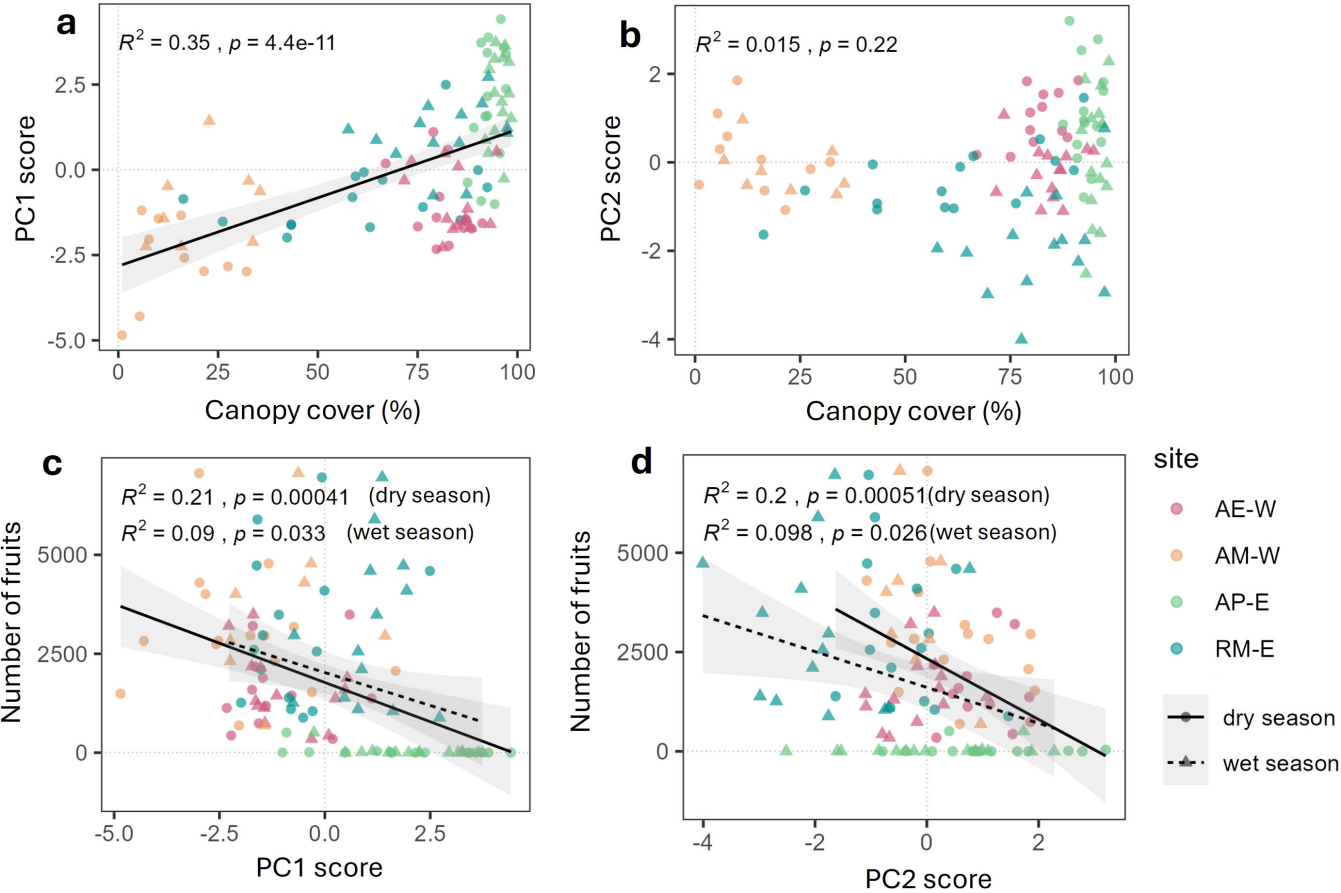
Relationship between principal component (PC) scores, environment, and reproductive output. **(a)** Linear relationship between PC1 score and canopy cover aggregated across seasons, **(b)** PC2 score vs canopy cover, **(c)** Linear relationship between fruit and PC1 score, **(d)** negative linear relationship between fruit and PC2 score. The data points refer to plant-level observations, where different colors represent sites and different shapes refer to seasons (circle for the dry season and triangle for the wet season). For **(c, d)**, a solid regression line is fitted to the dry season observation, while a dashed regression line is fitted to the wet season observation.

To understand the drivers for the differences in trait strategies between sites, we evaluated the relationship between canopy cover of individual coffee plants and their PC1 scores (**Figure 6**). Canopy cover correlates positively with PC1 scores when data are aggregated across all sites and seasons (R^2^=0.35, p<0.05; **Figure 6**). This indicates that coffee trees grown under higher canopy cover tend to have higher *A_mass_* and *V_cmax_*, but lower LMA and LDMC. PC2 scores, however, have no significant relationship with canopy cover.

We also evaluated the relationship between PC scores and the estimated number of fruits of individual coffee plants. The number of fruits is negatively correlated to both seasons’ PC1 score, although the relationship is stronger between fruit and the dry season PC1 score (R^2^=0.21, p<0.05). This indicates that coffee plants with higher *A_mass_* and *V_cmax_* but lower LMA and LDMC tend to have fewer fruits. Similarly, the number of fruits also correlates negatively with the dry and wet season PC2 scores (R^2^=0.098–0.2; p<0.05). This indicates that plants with higher *g_sw_* but lower leaf chemical traits are more likely to have less fruit.

## 4 Discussion

Using field measurements of coffee functional traits across sites and seasons in Java Island, Indonesia, our study revealed a clear spatial and seasonal variation in leaf traits of coffee plants (arabica, hybrid, and robusta). Among all traits, leaf physiological traits have the largest within-site variations, while structural traits have the least within-site variation with clear between-site differences. Across the temporal scale, photosynthetic capacity exhibited pronounced seasonality at a robusta site, whereas arabica and hybrid sites tend to show greater seasonality in leaf structural traits with no considerable changes in photosynthetic capacity. We also demonstrate the linkage between trait variation and shade and reproductive output, where denser shade promoted resource-acquisitive traits (higher photosynthetic capacity, lower leaf mass per area ratio) in coffee but did not necessarily lead to higher number of fruits per plant.

### 4.1 Trait variation across sites

Leaf structural, physiological, and chemical traits in our study showed substantial differences across sites. In particular, we observed clear site-level differences in LMA, leaf area, *A_max_*, *V_cmax_*, and to a lesser extent N and P. The observed values broadly fall within the reported range for arabica and robusta coffee from other studies in South and Central America, Africa, and Thailand. For example, our average *A_max_* ranges between 4–7 µmol CO_2_ m^2^ s^−1^, within the reported range for arabica (3–9 µmol CO_2_ m^2^ s^−1^) (Bote and Jan, 2016; Rodrigues et al., 2016) and in the lower end for robusta (5–15 µmol CO_2_ m^2^ s^−1^) (Rodrigues et al., 2016; Chiarawipa et al., 2025). Our leaf N (2.55–3.18%) is at the lower end of the reported range in arabica (2.1–4.4%) (Martin and Isaac, 2021; Gagliardi et al., 2023), possibly due to lower fertilizer inputs and nutrient availability. Arabica leaves showed the highest LMA (~113.27 g m^-2^), followed by the hybrids and robusta (81.52–94.7 g m^−2^), all within the reported range of both species (arabica: 36–110 g m^−2^, robusta: 78–93 g m^−2^) (Bote and Struik, 2011; Rodríguez-López et al., 2013; Buchanan et al., 2019; Gagliardi et al., 2023). *V_cmax_* and *J_max_* at our robusta site are within the range of reported values of 8.7–109 µmol CO_2_ m^2^ s^−1^ for *V_cmax_* and 30–102 µmol m^2^ s^−1^ for *J_max_* (Rodríguez-López et al., 2013; Chiarawipa et al., 2025). These comparisons suggest that while our systems fall within global trait spectra, they may be constrained at the lower end by nutrient and management limitations.

We found that structural traits showed low overall variation among individuals, while chemical and physiological traits showed greater variability. This pattern reflects how traits respond to environmental heterogeneity: structural traits are less sensitive to variation in environmental conditions because they are largely determined during leaf development and thus more costly (Grime and Mackey, 2002; Stotz et al., 2021); chemical traits respond to moderate variability in soil condition; and physiological traits are sensitive to the spatial variability of microclimate (Martin et al., 2017; Odé et al., 2025). At the site level, trait variation was generally low, consistent with other studies on arabica coffee, where homogenous management constrains divergence among individuals compared to natural systems (Martin et al., 2017; Buchanan et al., 2019). However, notable exceptions exist. For example, our *A_mass_* variability was the highest for site AM-W, which has the least shade, in contrast to Buchanan et al. (2019), who found the highest *A_mass_* variability under permanent shade. This is likely related to the more heterogeneous light availability at our site due to the varying canopy cover (31.18 ± 6.13%), despite the low mean shade.

### 4.2 Varying seasonality patterns in structural and physiological traits

Studies on evergreen species show mixed evidence of trait seasonality, with certain traits exhibiting larger variation between seasons than others. At the species level, Gotsch et al. (2010) reported an average decrease in LMA and an inconsistent change in leaf N in the wet season, whereas at the community level Bloomfield et al. (2018) found that seasonality explained a substantial proportion of variation in *V_cmax_* but not in LMA and leaf N. Such responses are often species-specific, and in coffee, previous studies have demonstrated an apparent seasonal shift in photosynthetic traits of both arabica and robusta (Silva et al., 2004; Baliza et al., 2012; Rodrigues et al., 2016).

In our study, seasonal patterns of trait variations differed between coffee types. At the robusta site (RM-E), *A_max_* and *V_cmax_* increased in the wet season, accompanied by an increase in leaf P, and no detected changes in leaf N and LMA. The positive correlation between *A_max_* and leaf P (but not leaf N) suggests that photosynthetic capacity may be P-limited. This limitation may be exacerbated by reduced P uptake under RM-E’s particularly low dry-season soil moisture (Radersma et al., 2005). Additionally, the influence of P on photosynthetic traits has been highlighted by cross-species studies, which reported a larger sensitivity of *A_max_* and *V_cmax_* to leaf N under higher P (Reich et al., 2009; Walker et al., 2014).

In contrast, our arabica and hybrid sites showed no changes in photosynthetic traits between seasons. Instead, we observed a decrease in structural traits (LMA, leaf area, and leaf thickness) accompanied by a decrease in leaf N during the wet season. Higher LMA in drier conditions is commonly linked to thicker leaf cell walls to reduce transpiration (Matos et al., 2009; Buchanan et al., 2019). Accordingly, lower LMA in the wet season is accompanied by lower leaf N, since N makes up a substantial proportion of the cell wall (Onoda et al., 2017). The decrease in leaf N could also reflect translocation of photosynthates to fruits in the wet season, which coincides with the fruit-filling stage.

The absence of *A_max_* seasonality at our arabica sites contrasts with findings from Southeastern Brazil, where both arabica (Silva et al., 2004; Baliza et al., 2012; Rodrigues et al., 2016) and robusta (Rodrigues et al., 2016) show higher *A_max_* during the wet season. These contrasting results likely reflect differences in seasonal climate drivers: in Southeastern Brazil, the wet season coincides with higher radiation and warmer temperatures, whereas in Java the wet season brings cooler, cloudier conditions that may suppress photosynthesis despite abundant water.

### 4.3 Trait trade-offs along the leaf economic spectrum and nutrient-stomatal axis

More than half of the trait variation in our coffee plants was captured in a two-dimensional plane, with the primary axis aligning closely with the Leaf Economic Spectrum (LES) framework (Wright et al., 2004). One end of the LES represents coffee plants with a quick return on investment or ‘resource-acquisitive’ plants, as noted by the high photosynthetic capacity, and low LMA and LDMC (positive PC1). The other end of the LES represents plants with slow return of investment or ‘resource-conservative’ plants, as noted by the high LMA and LDMC, and low photosynthetic capacity (negative PC1) (**Figure 5**). PC2 represented a nutrient-stomatal trade-off, contrasting plants with high *g_sw_* and photosynthetic capacity but low nutrient, against those with lower *g_sw_* and photosynthetic capacity but higher nutrient.

**Figure 7:**
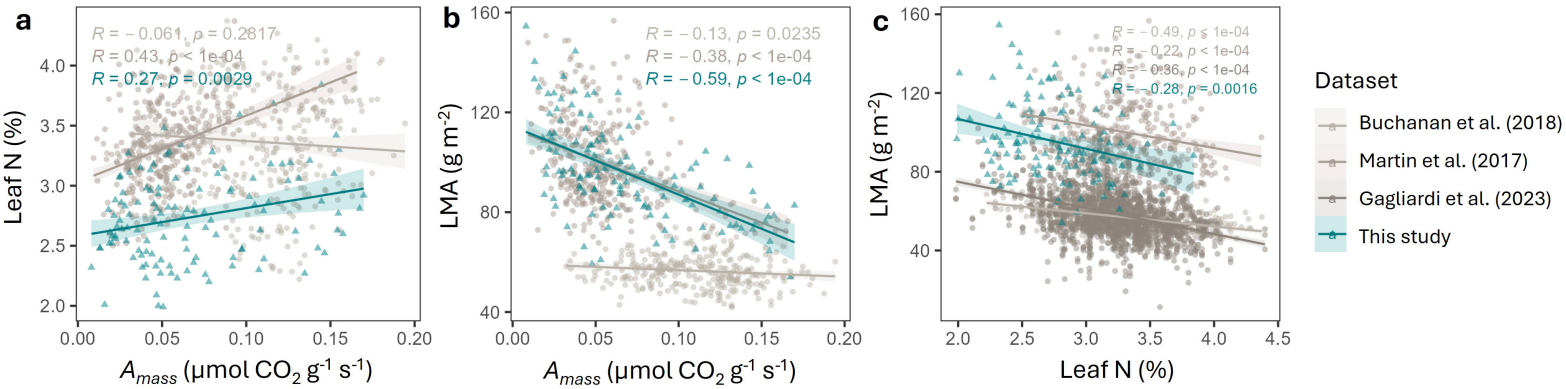
Pairwise trait relationship of **(a)** Leaf nitrogen (N) content vs. light-saturated photosynthetic rate per unit dry mass (A_mass_), **(b)** leaf mass per area (LMA) vs. A_mass_, **(c)** LMA vs. leaf N content. Grey datapoints are observations obtained from the TRY database, and the blue triangle data points are observations from this study aggregated across sites and seasons.

Pairwise trait correlations among leaf traits (leaf N – *A_mass_* – LMA) of coffee in our study largely follow the global pattern of LES of natural plants (Wright et al., 2004) and that of other coffee studies (Martin et al., 2017; Buchanan et al., 2019; Gagliardi et al., 2023) (**Figure 5**). As predicted by LES, leaf N was positively correlated with A_mass_, since much of leaf N is invested in Rubisco. However, the N–A_mass_ relationship (r=0.27, p< 0.05) was weaker than in the global LES (**Figure 7a**), consistent with previous reports for coffee (Wright et al., 2004; Martin et al., 2017; Buchanan et al., 2019). A substantial proportion of leaf N may reside outside photosynthetic enzymes or cell walls, for example, in alkaloids (Onoda et al., 2017) such as caffeine. In coffee, N residing in caffeine constitutes approximately 0.35 to 0.46% of the leaf dry weight (Gonthier et al., 2011; Vega et al., 2020), which corresponds to 11-20% of total leaf N. Additionally, caffeine content is often higher in shade-grown coffee plants (Vaast et al., 2006), which may further decouple leaf N from leaf *A_mass_*. We also found a strong negative LMA–*A_mass_* relationship (r=-0.59, p< 0.05, **Figure 7b**) and a negative LMA–leaf N relationship (r=-0.28, p< 0.05, **Figure 7c**), which aligns well with LES prediction. Higher LMA is associated with a higher proportion of cell walls relative to leaf dry mass, which could limit photosynthesis by limiting CO_2_ diffusion to chloroplasts and/or reallocating N to cell walls instead of Rubisco (Onoda et al., 2017). Overall, our LMA–N–*A_mass_* relationships are more consistent with Martin et al. (2017) than Buchanan et al. (2019). One possible explanation is that the fertilization treatments from Buchanan et al. (2019) resulted in higher and less variable leaf N compared to our sites that are less-fertilized, which may have weakened their trait relationship relative to the LES pattern of natural ecosystems.

### 4.4 Denser shades are associated with a resource-acquisitive trait strategy

There are two hypotheses generally used to explain plant strategies to tolerate shade, which are (1) increasing investment in storage and defence (resource-conservative), or (2) maximizing carbon gain (resource-acquisitive) (Valladares et al., 2016). Shade-gradient studies in coffee agroecosystems reported conflicting findings, with some supporting the first hypothesis (Martins et al., 2014; Buchanan et al., 2019; Getachew et al., 2022), and the rest supporting the latter (Freitas et al., 2003; Bote and Struik, 2011; Gagliardi et al., 2015). Along the LES axis, we found that higher canopy cover, which likely limits light availability, is associated with resource-acquisitive traits in coffee plants, aligning with the second hypothesis. Shading may influence traits indirectly via microclimate buffering (Piato et al., 2020). For instance, higher humidity in the understory (Oliosi et al., 2016) could increase *g_sw_* and subsequently increase photosynthesis and/or decrease LMA of coffee plants (Bote et al., 2018). Nevertheless, the effect of canopy cover on leaf traits is complex and may not be explained by light availability alone, with some studies reporting a non-linear increase in photosynthesis with increasing shade (Baliza et al., 2012).

In terms of reproductive output, we found that resource-acquisitive plants tend to have fewer fruits. This pattern aligns with previous studies that reported lower fruit count in coffee plants with higher leaf N and *A_mass_* and lower LMA (Gagliardi et al., 2015; Bote and Jan, 2016). This inverse relationship between leaf carbon gain and fruits may be a result of the trade-off between vegetative and reproductive growth, as previously noted (Rodrigues et al., 2016; Piato et al., 2020). Meanwhile, along the nutrient-stomatal axis, we found that plants with higher *g_sw_* and C content, coinciding with lower nutrients, tend to bear more fruits. Phenological studies explain the nutrient-fruit relationship through nutrient translocation, where photosynthetic products from the leaves are translocated to developing fruits, thus lowering leaf N, P, and K during the fruit-filling phase (León-Burgos et al., 2024b; Oliosi et al., 2020; Santos et al., 2021; Silva et al., 2022). While previous studies reported an increase in photosynthesis at a later fruiting stage due to rising carbon demand for fruit development (León-Burgos et al., 2024b), DaMatta et al. (2008) argued that this response is mainly due to changes in *g_s_*, although the mechanism is not yet well understood. Other more direct physiological factors on flowering and fruit development are hormonal signalling (Sadka et al., 2023) and resource allocation/mass partitioning between vegetative and reproductive organs (León-Burgos et al., 2024a).

However, we did not observe a direct relationship between canopy cover and fruit count at our sites. Instead, the relationship is likely to be indirect, where: greater shade → more resource-acquisitive plants → fewer fruits. The effect of shade on fruit is often inconsistent, where some studies observed an increased productivity with increasing shade (Vaast et al., 2006), whereas others observed no effect (Oliosi et al., 2016). Overall, the effect of shade on growth and fruit appears to be complex, depending on factors such as plant age, cultivar, and other site conditions (Piato et al., 2020). It is also important to note that our estimates of fruit count do not capture total yield, bean size, bean quality, and multiple fruiting periods. These aspects can substantially influence yield outcomes, for example, due to planting density and the trade-offs between fruit loads and bean size (Vaast et al., 2006).

Additionally, while we assessed shading levels through canopy cover, we did not specifically look into the effects of shade tree species composition. Shade species can differ in their canopy architecture (compact vs. diffuse canopy, leaf angle, and leaf area), thus having different influence in microclimate buffering (e.g., light interception, wind buffering) and pathogen transmission (Gagliardi et al., 2020, 2023). Shade tree species also vary in their leaf traits such as leaf N, C, lignin, and phenols, leading to differences in litter decomposition, and consequently soil nutrient and water cycling (Isaac et al., 2024; Misgana et al., 2024). These shade species-specific effects are not captured by canopy cover measurements alone and thus could contribute to some of the site-specific trait variations we observed.

### 4.5 Trait-based perspectives for the coffee agroecosystem

Trait coordination in agricultural plants is often weaker than natural plants, likely due to selection breeding for specific domestication traits (e.g., seed size) under an environment with abundant resources (Milla et al., 2014). Cultivation is hypothesized to lead to plants with a more resource-acquisitive trait strategy compared to their progenitor (Milla et al., 2015), but this has not been studied for coffee cultivars. Focusing breeding efforts for optimizing productivity and climate resilience will require a better understanding of coffee plant trait trade-offs and plasticity across genotypes and environments. While the effects of genotypes on trait trade-offs cannot be disentangled in our study, we provide a first step toward future comparative analysis.

Beyond site-level insights, the observed variations in traits provide valuable parameters for constraining process-based models of coffee productivity and carbon cycling (Van Noordwijk and Lusiana, 1999; van Oijen et al., 2010; Vezy et al., 2020). These models consider competition for light, water, and nutrients, thus can be used to assess the optimum management treatments (e.g., planting density, thinning, fertilization) that can maximize yield with minimum environmental impacts (van Oijen et al., 2010). Integrating traits into modelling frameworks can support sustainable coffee management and climate-smart agriculture by helping farmers design management strategies before implementing them in the field.

## 5 Conclusion

Our study revealed clear site-level differences in structural, chemical, and photosynthetic traits across Indonesian coffee agroecosystems. Photosynthetic traits exhibited pronounced seasonality at the robusta coffee site, whereas arabica coffee and hybrid coffee sites tend to show less variation in photosynthetic traits but greater seasonal variation in leaf structural traits. Furthermore, denser shade conditions promoted coffee to have resource-acquisitive traits (higher photosynthetic capacity, lower LMA) but did not necessarily result in higher fruit production. Our finding highlights the variation in the ecophysiology of coffee across sites, but quantifying the contribution of each factor (i.e., genotype, fertilization treatment, climate) might require broader sampling. Overall, our study provides one of the first field-based assessments of plant functional traits of coffee agroecosystems in Indonesia, and provides the critical information to understand the ecophysiology of coffee and the impacts of coffee expansion on the regional carbon cycle.

## Data Availability

The raw data supporting the conclusions of this article will be made available by the authors, without undue reservation.
