# Supplementary material for: Characterizing the plant functional traits of coffee agroecosystems in Indonesia
Source: Front Plant Sci. 2026 Jan 19;16:1743035. doi: 10.3389/fpls.2025.1743035 (PMC12861878; doi:10.3389/fpls.2025.1743035)
Supplement: Supplementary file 1 [file DataSheet1.docx]

Supplementary Material

# Supplementary Figures


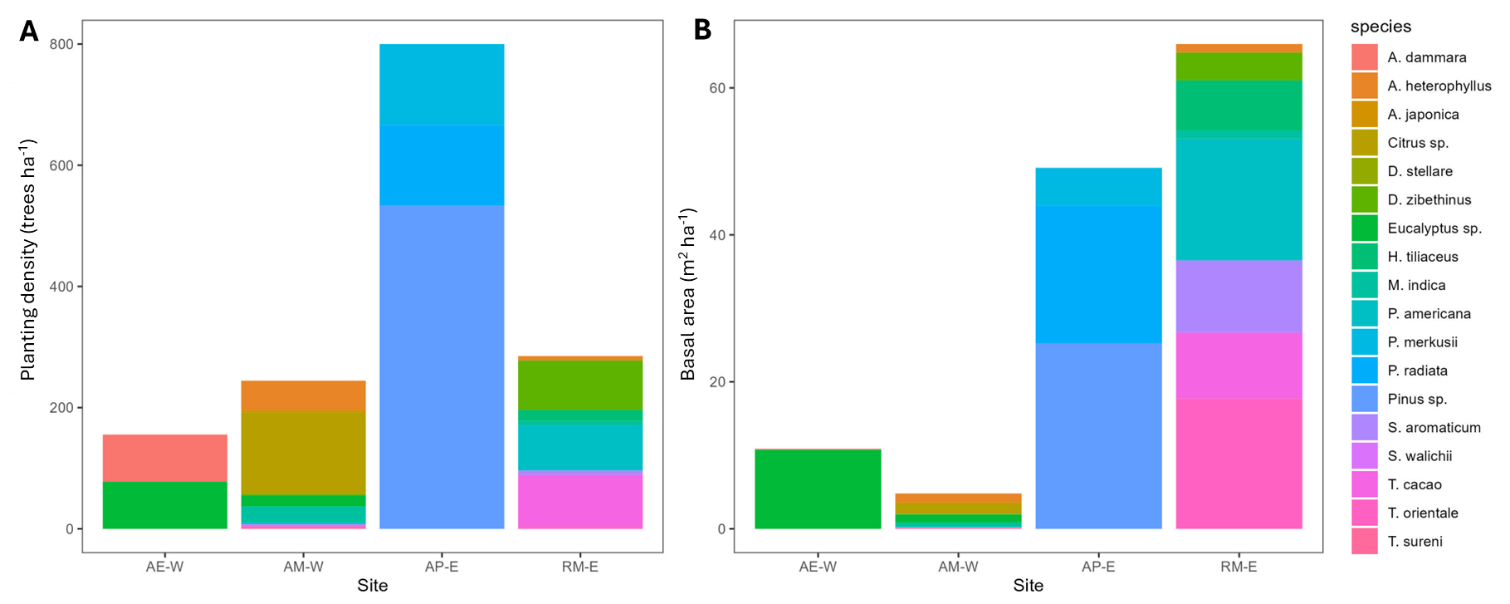


**Supplementary Figure 1.** Characteristics of shade trees across sites. **(A)** planting density (trees ha^-1^), **(B)** basal area (m^2^ ha^-1^).


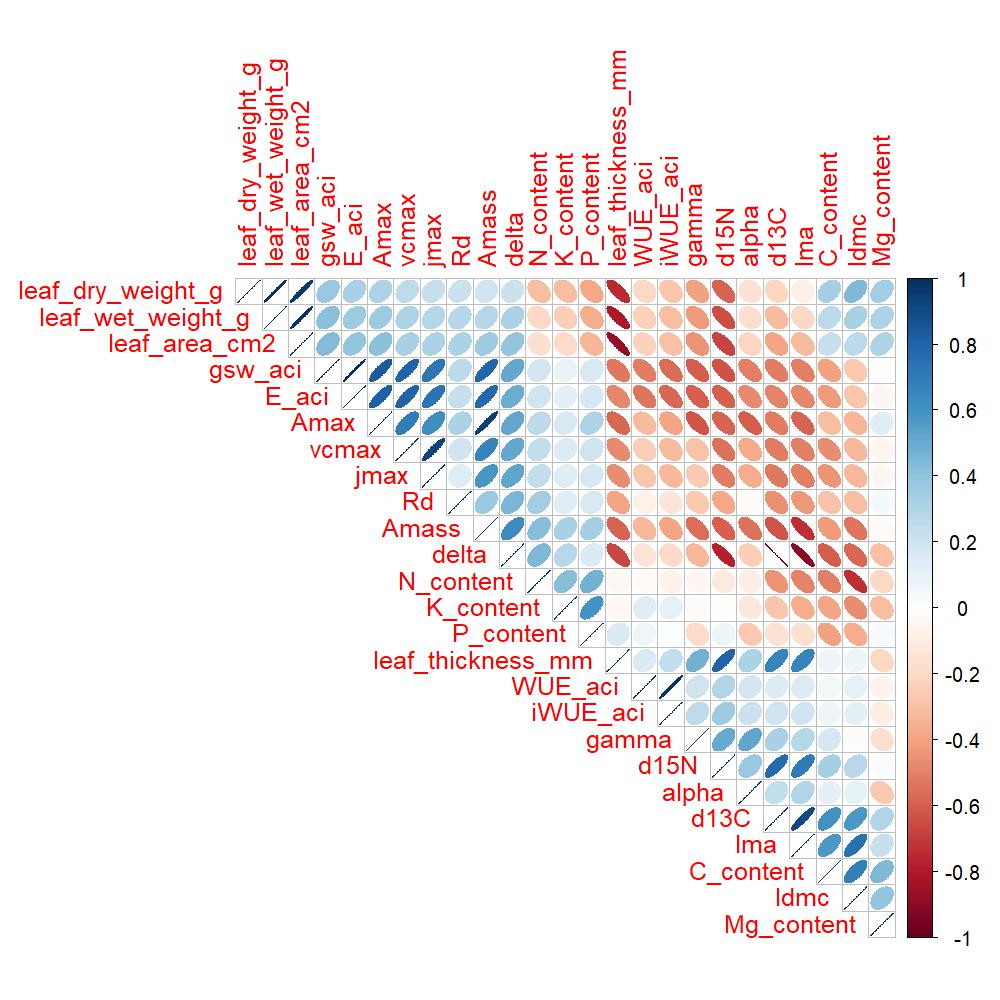


**Supplementary Figure 2.** Pairwise correlation between leaf traits. Color bar refers to the pearson correlation coefficient.


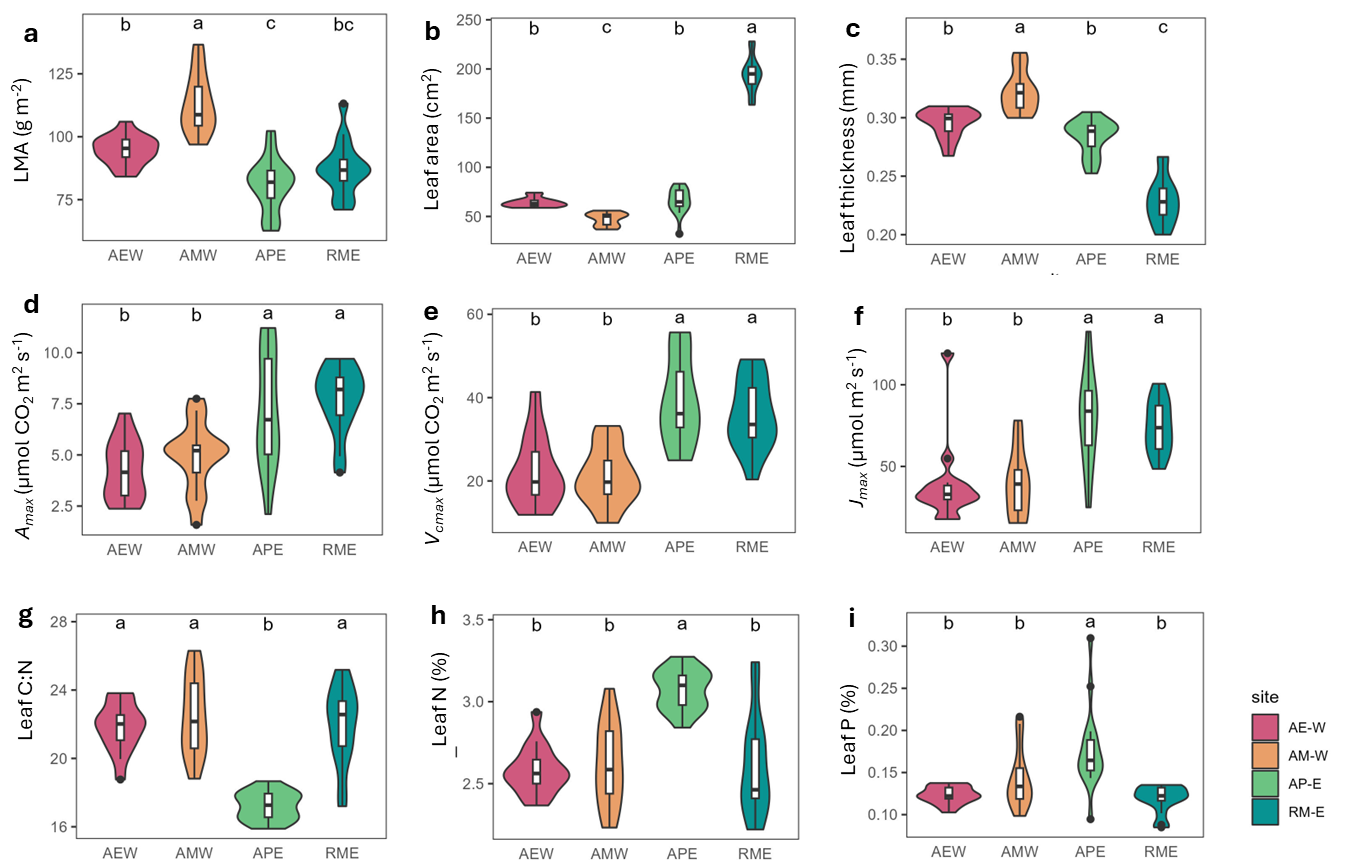


**Supplementary Figure 3.** Leaf structural, physiological, and chemical traits across different sites. **(A)** leaf mass area, **(B)** leaf area, **(C)** leaf thickness, **(D)** light-saturated photosynthetic rate per unit leaf dry mass (A_mass_), **(E)** maximum carboxylation rate (V_cmax_), **(F)** maximum electron transport rate (J_max_), **(G)** leaf C:N, **(H)** leaf nitrogen content, and **(I)** leaf phosphorus content. Different colors of the violin plot refer to different sites. Letters above the violin plot indicates significantly different means following Tukey HSD test, where groups that differ significantly will have different letters and groups that do not differ from each other share the same letter.
